# Clinical Impact of Vaping

**DOI:** 10.3390/toxics13060470

**Published:** 2025-06-01

**Authors:** Francesco Petrella, Paola Faverio, Andrea Cara, Enrico Mario Cassina, Lidia Libretti, Sara Lo Torto, Emanuele Pirondini, Federico Raveglia, Francesca Spinelli, Antonio Tuoro, Elisa Perger, Fabrizio Luppi

**Affiliations:** 1 Department of Thoracic Surgery Fondazione IRCCS san Gerardo dei Tintori, 20900 Monza, Italy; andrea.cara@irccs-sangerardo.it (A.C.); enricomario.cassina@irccs-sangerardo.it (E.M.C.); lidia.libretti@irccs-sangegardo.it (L.L.); sara.lotorto@hotmali.it (S.L.T.); emanuele.pirondini@irccs-sangerardo.it (E.P.); federico.raveglia@irccs-sangerardo.it (F.R.); francesca.spinelli@unimi.it (F.S.); antonio.tuoro@irccs-sangerardo.it (A.T.); 2 Department of Respiratory Diseases Fondazione IRCCS san Gerardo dei Tintori, Monza (MB), Italy and University of Milano Bicocca, 20126 Milan, Italy; paola.faverio@unimib.it (P.F.); elisa.perger@unimib.it (E.P.); fabrizio.luppi@unimib.it (F.L.); 3 Istituto Auxologico Italiano IRCCS San Luca Hospital, Department of Cardiology, Sleep Center, 20126 Milan, Italy

**Keywords:** vaping, electronic cigarette, e-smoking, lung cancer, chronic obstructive pulmonary disease (COPD), asthma

## Abstract

The term ‘vaping’ refers to the use of electronic cigarettes or other devices to inhale a variety of heated and aerosolized substances. Vaping has been promoted as a less harmful and potentially oncogenic alternative to nicotine cigarettes, particularly to help heavy smokers quit. While vaping products do not produce the same carcinogenic substances—such as polycyclic aromatic hydrocarbons—generated by the combustion of tobacco, and while their fluids lack tobacco-related carcinogens like nitrosamines, it is now well established that they still generate harmful and potentially oncogenic byproducts. Several mechanisms have been proposed to explain the potential oncogenic effects of vaping fluids, including direct chemical action, epithelial–mesenchymal transition induction, redox stress, mitochondrial toxicity, and DNA damage. In addition to cancer risk, there have been reports of adverse effects on cardiovascular health, reproductive function, and non-oncologic lung injuries. These include exogenous lipoid pneumonia, diffuse alveolar hemorrhage with proven alveolar injury, and vaping-associated bronchiolitis obliterans. The aim of this review is to examine vaping devices, their potential role in lung carcinogenesis, vaping-associated lung injury, and other clinical implications, including impacts on cardiovascular, cerebrovascular, and respiratory diseases, and also pregnancy and fetus health.

## 1. Introduction

The term “Electronic Nicotine Delivery System” (ENDS) was first introduced by the World Health Organization (WHO) in 2009 to describe the various types of electronic cigarettes and vaping devices that deliver nicotine [1], which emerged in the international market in 2006 [2]. The term “vaping” is commonly used to refer to the practice of using e-cigarettes. The terms “vaping device”, “e-cigarette”, and “electronic cigarette” are synonyms and refer to devices that heat e-liquid, producing an aerosol (vapor) that is inhaled by the user. These devices have been promoted as a less harmful alternative to traditional cigarette smoking and as a practical tool to help heavy smokers quit. However, vaping has become increasingly popular among young adults (18–24 years old) and adolescents (11–17 years old), particularly among those who have never smoked. This rise in popularity is largely due to the misconception that vaping has no adverse effects on health. As a result, it has often led never-smokers to experiment with smoking, rather than helping established smokers quit. Furthermore, vaping devices are not limited to nicotine; they also offer a variety of flavor options, including marijuana and concentrated tetrahydrocannabinol (THC), the use of which has significantly increased among adolescents [3]. Given the widespread use of vaping among young people and the risk of nicotine addiction, coupled with the incomplete understanding of its clinical impacts, there is an increasing body of literature focused on the health effects of electronic smoking. This review aims to examine vaping devices, their potential role in lung carcinogenesis, vaping-associated lung injuries, and other clinical implications for cardiovascular, cerebrovascular, and respiratory diseases. We included in our review English-language papers focusing on vaping, e-cigarettes, oncology, and pneumology, published between 2002 and 2025.

## 2. Vaping: How It Works

E-cigarettes are electronic devices powered by batteries. They consist of an atomizer—also known as a heating element—that heats the e-liquid to generate vapor, and a cartridge containing the e-liquid [4]. Typically, the e-liquid contains flavorings and humectants, with or without nicotine. The atomizer heats the e-liquid, producing an aerosol (vapor) that is inhaled by the user. Since there is no direct combustion of tobacco or other carcinogenic materials found in traditional cigarettes—such as paper and tobacco—it was initially believed that vaping devices had no adverse effects on human health. However, it is now well established that the heating of various compounds can lead to the formation of potentially harmful chemicals [5,6]. To date, four generations of e-cigarettes have been developed. The first generation resembled traditional cigarettes in appearance. The second generation, known as “clearomizers”, featured a larger detachable reservoir for e-liquid, a multi-voltage battery, and a removable filament. The third generation, referred to as “mods”, introduced modified batteries with adjustable voltage, wattage, and power settings. The fourth—and currently most recent—generation includes devices resembling USB flash drives, which use fixed-voltage batteries and come in various shapes and sizes [7]. Nicotine is one of the most commonly used components in e-liquids. Initially, nicotine concentrations ranged from 3 to 36 mg/mL. Today, nicotine salts—compounds formed by lowering the pH of free-base nicotine with acids such as lactic, tartaric, salicylic, or benzoic acid—are more commonly used. These can contain over 50 mg/mL of nicotine. Notably, the aerosolization of nicotine alters its pharmacokinetic profile, potentially resulting in long-term health effects that are not yet fully understood. Specifically, the pH of e-cigarette aerosols can affect nicotine’s bioavailability and absorption rate, potentially leading to faster nicotine delivery and reduced vaping frequency [7]. A variety of compounds are used to flavor e-liquids. Vanillin, ethyl vanillin, ethyl maltol, and menthol are just a few examples of flavoring agents that may cause cytotoxicity, irritation, inflammation, oxidative stress, and respiratory diseases [8]. Humectants—such as propylene glycol and glycerol—act as carriers for nicotine and flavorings during aerosolization. Several studies have reported acute toxicity, skin and respiratory irritation, and physiological alterations following exposure to these compounds [8,9]. Heating elements in vaping devices can also release metals such as cobalt, nickel, selenium, titanium, manganese, molybdenum, chromium, and aluminum. Vaping temperatures typically remain below 300 °C as long as the wick remains saturated with e-liquid. However, when the wicking material dries out, temperatures can exceed 1000 °C, accelerating coil degradation and metal release. Vaping also generates various thermal degradation products. For instance, at 157 °C, propylene glycol oxidizes to form formaldehyde, acetone, and acetaldehyde—compounds known to be carcinogenic [3].

## 3. Molecular Mechanisms of Vaping-Induced Damage and Potential Role in Lung Carcinogenesis

### 3.1. Nicotine

Metabolic byproducts resulting from high levels of inhaled nicotine can induce toxicity. Nicotine is primarily metabolized into nicotine iminium, which is further processed by aldehyde oxidase to form cotinine. It has been demonstrated that nicotine iminium, in the presence of aldehyde oxidase, is a significant trigger for superoxide generation. Furthermore, aldehyde oxidase is normally expressed in the lungs, and chronic exposure to vaping in mice has been shown to significantly increase its expression. Nicotine and its metabolites enhance superoxide production in the lungs—a process that is inhibited when aldehyde oxidase is blocked. In summary, nicotine metabolism stimulates aldehyde oxidase-mediated superoxide generation, which contributes to lung damage. Therefore, high and uncontrolled levels of nicotine inhalation, such as those associated with vaping, can result in oxidative lung injury [10,11,12].

### 3.2. Tetrahydrocannabinol (THC)

Cannabinoids are frequently present in e-cigarettes, either alone or in combination with other substances that may exacerbate lung injury. Early reports of E-cigarette or Vaping Product Use-Associated Lung Injury (EVALI) frequently noted the co-presence of tetrahydrocannabinol (THC) and vitamin E acetate (VEA) in patients with more severe lung damage, suggesting that the vast majority—but not all—of EVALI cases were closely linked to this combination. The wide variability in cannabinoid content and additive concentrations poses a challenge in determining the specific role of individual components in EVALI pathogenesis. While a definitive THC concentration threshold for increased EVALI risk has not been identified, it is now recognized that certain components within cannabinoid-containing vape liquids may be more harmful to pulmonary epithelium than nicotine. Additionally, several flavoring compounds used in vape liquids—such as diacetyl and 2,3-pentanedione—can impair bronchial ciliary function and alter gene expression in bronchial epithelial cells [13].

### 3.3. Flavoring Agents

The most commonly used flavoring agents in e-cigarettes include diacetyl, acetoin, p-menthone, triacetin, ethyl maltol, triethyl citrate, 3-hexen-1-ol, α-terpineol, methylanthranilate, benzyl alcohol, perillartine, vanillin, methyl dihydrojasmonate, melonal, and γ-decalactone [14]. Flavoring compounds such as diacetyl, 2,3-pentanedione (also known as acetylpropionyl), o-vanillin, and maltol have been shown to induce the production of reactive oxygen species (ROS) and interleukin-8 (IL-8), thereby promoting inflammation and impairing lung function. Menthol, commonly added to tobacco products to mask bitterness and enhance user tolerance, also contributes to harm. Its antipruritic properties and ability to prolong breath-holding increase the retention and absorption of cytotoxic components in e-cigarette aerosol. Moreover, menthol exposure is significantly associated with oxidative damage to mitochondrial proteins, reduced mitochondrial function, increased pro-inflammatory signaling, and inhibition of pulmonary surfactant production [14]. Humectants in e-cigarettes, like propylene glycol and vegetable glycerin, are hygroscopic substances that retain moisture, representing one of the main components of e-liquid. These humectants are used to generate the aerosol and act as a carrier for nicotine and flavorings. Propylene glycol is metabolized in the liver by alcohol dehydrogenase to form metabolites like lactaldehyde, which is then further converted to lactic acid (lactate) and pyruvate. A portion is also excreted unchanged by the kidneys. High doses of propylene glycol can lead to metabolic acidosis and other toxic effects [14].

## 4. Vaping and Cancer

A recent systematic review of thirty-nine studies—including two longitudinal observational studies, nine cross-sectional studies, one case report, and twenty-seven in vitro and animal studies—investigated the relationship between e-cigarette use and the risk of lung or other types of cancer. Although no significant increase in cancer risk was observed among never-smoker current vapers, there was strong evidence linking vaping exposure to cellular apoptosis, oxidative stress, DNA damage, genotoxicity, and tumor growth, particularly following acute exposure. These findings indicate substantial evidence that vaping is associated with biomarkers predictive of cancer risk [15].

### 4.1. Lung Cancer

Many longitudinal and cross-sectional studies have not demonstrated a significant association between vaping and lung cancer. However, studies employing alternative methodologies often support a suspected increased risk. One longitudinal study involving 119,593 participants found that individuals diagnosed with lung cancer were more frequently former or current vapers, including “dual users”—those using both e-cigarettes and traditional cigarettes. Nonetheless, this study did not identify a significant relationship between vaping and the incidence of lung cancer [16]. A study assessing salivary DNA methylation biomarkers for lung cancer also did not find an elevated risk among never-smoker current vapers [17]. In contrast, another study reported accelerated pulmonary decline in non-smoker current vapers compared to never-smokers, suggesting an elevated risk of age-related pulmonary diseases, including neoplastic conditions [18]. Preclinical studies have reported a marked decrease in cell viability and increased apoptosis following acute exposure to both nicotine and non-nicotine e-cigarettes [19,20]. Additionally, significant oxidative stress and elevated biomarkers were observed after acute and short- to medium-term nicotine e-cigarette exposure [21]. Findings on genotoxicity and mutagenic protein expression remain mixed: some studies observed increases following acute nicotine exposure, others after short- to medium-term exposure, while some reported no significant genotoxic effects at all [22,23,24]. Multiple animal studies involving both male and female mice confirmed significant oxidative stress associated with nicotine e-cigarette use, suggesting an increased predisposition to lung cancer [25,26]. Lung cancer—across its various histological subtypes and genomic profiles—remains the leading cause of cancer-related deaths worldwide. Thus, primary prevention through risk factor reduction should be strongly encouraged by national health authorities [27,28,29,30]. Currently, there is no specific lung cancer screening program targeted exclusively at individuals who use e-cigarettes. Lung cancer screening programs are primarily focused on current or former smokers aged between 50 and 80 years with a smoking history of at least 20 pack-years. Therefore, former traditional smokers who have transitioned to vaping products, as well as dual users, may be eligible for these programs. However, more robust evidence might, in the future, support the extension of preventive screening programs to e-cigarette users and individuals with other relevant environmental exposures. It is worth noting that, according to a recent cross-sectional study conducted in the United States involving 22,713 individuals eligible for lung cancer screening, e-cigarette use was independently associated with a lower likelihood of undergoing screening, particularly among individuals who had quit smoking traditional cigarettes [31].

### 4.2. Other Types of Cancer

Although only one of the longitudinal and cross-sectional studies included in the aforementioned review reported an increased risk of cancer associated with vaping, most studies employing other methodologies found a significantly elevated cancer risk following e-cigarette exposure. Chen et al. observed significantly lower levels of urinary carcinogens in exclusive e-cigarette users compared to current tobacco smokers and also reported higher levels of acrolein metabolites in non-smoker current vapers compared to never-vapers [32]. Acrolein, an unsaturated aldehyde, is associated with respiratory tract carcinogenesis via oxidative DNA damage, and the World Health Organization recommends limiting its intake to no more than 7.5 µg/day per kg of body weight [33,34,35]. Three cross-sectional studies found no significant association between vaping and non-melanoma skin cancer, bladder cancer, or overall cancer incidence [36,37,38]. However, Wharram et al. reported higher odds of e-cigarette use among female patients with metastatic breast cancer compared to those with non-metastatic disease. This association was not observed in colorectal or prostate cancer [39]. Other studies have shown that non-smoker current vapers exhibit higher levels of inflammatory and cancer-associated salivary biomarkers than never-smoker non-vapers, although these levels were still lower than those found in current tobacco smokers [40]. Similarly, another study found reduced levels of DNA damage markers in the oral cells of non-smoker vapers compared to tobacco smokers [41]. Several preclinical studies have demonstrated that acute exposure to vaping accelerates the progression of various cancers—including brain tumors, bladder cancer [27], and oral squamous cell carcinoma [18,32]—and one study specifically reported significantly faster cerebral tumor growth following e-liquid exposure [42,43].

## 5. Non-Oncologic Vaping-Associated Lung Injuries

E-cigarette use has been associated with several health-related harms [44,45]. Although the literature on the respiratory effects of e-cigarette use is still evolving, existing evidence has identified both short- and long-term pulmonary risks [46]. Meta-analyses indicate that, while some studies suggest a lower risk of respiratory disease from e-cigarettes compared to combustible cigarettes, other studies report comparable levels of respiratory impairment [47].

### 5.1. Chronic Obstructive Pulmonary Disease (COPD)

Chronic obstructive pulmonary disease (COPD) is a heterogeneous lung condition characterized by airway (bronchitis) and/or alveolar (emphysema) abnormalities. It typically presents with persistent and progressive airflow limitation, leading to symptoms such as dyspnea, chronic cough, sputum production, and exacerbations [48]. Traditionally, COPD has been associated with an abnormal inflammatory response to tobacco smoke in susceptible individuals [49], which accelerates age-related lung function decline and causes airway remodeling [50]. However, recent data indicate that up to one-third of COPD patients worldwide are non-smokers. Other environmental exposures, including biomass fuel smoke for cooking and heating [51] and urban air pollution [52] are now recognized as significant risk factors for COPD. Moreover, e-cigarette use is increasingly implicated in COPD development. A large cohort study found that e-cigarette use was independently associated with chronic bronchitis, emphysema, and COPD—even after adjusting for cigarette smoking [53]. Similarly, a meta-analysis of seven studies involving 3,552,424 individuals reported a 1.50-fold increased risk of COPD among e-cigarette users (95% CI: 1.27–1.73) [54]. E-cigarette use may also worsen pre-existing respiratory conditions and reduce the effectiveness of Continuous Positive Airway Pressure (CPAP) therapy in patients with co-existing sleep apnea [55]. Additionally, increased expression of matrix metalloproteinases has been reported in the sputum and airway biopsies of e-cigarette users [56,57]. Two animal studies showed histologic evidence of emphysema after 6 weeks and 4 months of exposure to aerosol from unflavored e-liquids [58,59]; in one case, emphysema was dependent on the presence of nicotine [58]. A third study found no emphysema after 4 months of exposure, likely due to a lower aerosol dose [60]. Furthermore, secondhand exposure to nicotine-containing vapor was associated with bronchitic symptoms and shortness of breath in non-users, with significant public health implications [61].

### 5.2. Asthma

Asthma is a chronic inflammatory disease of the airways, marked by variable airflow obstruction due to airway narrowing and wall thickening. It presents with symptoms such as wheezing, dyspnea, chest tightness, and cough [48]. While asthma is caused by a combination of genetic and environmental factors, common environmental triggers include tobacco smoke, allergens, and air pollution [62,63]. Smoking tobacco is known to worsen airway inflammation and exacerbate asthma symptoms [64,65]. With the growing use of e-cigarettes, attention has turned to their possible role in asthma. A meta-analysis of 10 cross-sectional studies in adolescents found an association between both current and lifetime e-cigarette use and asthma, independent of smoking status [66]. Similar findings have been reported in adults [67]. In animal models, e-cigarette aerosol has been shown to induce airway hyper-responsiveness [68], with increased bronchoconstriction in response to methacholine [69]. A single puff of e-cigarette aerosol was sufficient to activate vagal bronchopulmonary C-fibers and cause transient bronchoconstriction in anesthetized guinea pigs—effects reversed by anticholinergics [69]. Additionally, nicotine-containing aerosols altered mucin expression (notably MUC5AC) and increased airway eosinophilia, while nicotine-free aerosols did not [58]. These findings support the hypothesis that e-cigarette aerosol can provoke or exacerbate asthma and bronchoconstriction, particularly in patients with pre-existing airway diseases such as COPD. Further research is needed to clarify the role of nicotine and flavoring compounds in these responses.

### 5.3. EVALI

E-cigarette or Vaping Product Use-Associated Lung Injury (EVALI) is an acute or subacute respiratory illness with a broad spectrum of clinical and pathological features. According to the U.S. Centers for Disease Control and Prevention (CDC), diagnosis of EVALI requires recent (within 90 days) e-cigarette use, radiographic evidence of pulmonary infiltrates (on chest X-ray or CT scan), and the exclusion of other known causes such as infection [69]. EVALI primarily affects adolescents and young adult males, often following the use of THC- and vitamin E acetate-containing products from illicit sources [70,71]. Symptoms are non-specific and mimic other respiratory illnesses, including viral infections and diffuse alveolar damage. Imaging commonly shows bilateral ground-glass opacities, although other patterns—such as interstitial infiltrates, nodules, or pleural effusions—may also occur [72]. In a cohort of 304 patients, bronchoalveolar lavage (BAL) was negative for infectious agents, and cytology showed increased neutrophils, macrophages (often lipid-laden), and lymphocytes. Autopsy findings revealed diffuse alveolar damage with macrophages containing lipid and brown–black granular material, as well as hyperplasia of type 2 pneumocytes and atypical mitotic figures. Treatment primarily consists of supportive care, including oxygen supplementation or high-flow nasal cannula. In severe cases, mechanical ventilation is required (26% of cases), and ECMO is rarely necessary [73]. The exclusion of infectious etiologies is essential, and empiric antiviral or antibiotic treatment should be considered. Systemic corticosteroids may be beneficial in patients with severe EVALI and hypoxemia, although evidence is limited. The recommended dosing includes methylprednisolone 0.5–1 mg/kg/day or prednisone 40–60 mg tapered over 14 days [74,75].

### 5.4. Smoking-Related Diffuse Parenchymal Lung Diseases

Case reports and small case series have linked e-cigarette use to a variety of diffuse parenchymal lung diseases, including the following: small airway-centered fibrosis and constrictive bronchiolitis [76], lipoid pneumonia [77], diffuse alveolar hemorrhage [78], organizing pneumonia [79], respiratory bronchiolitis-associated interstitial lung disease [80], and hypersensitivity pneumonitis [81].

## 6. Vaping and Cardiac Diseases

Many of the substances contained in or produced by e-cigarettes—particularly nicotine, flavorings, propylene glycol, and glycerol—exert adverse cardiovascular effects. These include increased oxidative stress, inflammation, endothelial dysfunction, the promotion of atherosclerosis, platelet dysfunction, and hemodynamic changes [82]. Nicotine is the primary agent responsible for the cardiovascular harm linked to e-cigarette use. Animal studies have shown that nicotine-containing vape aerosols can impair myocardial function. A recent study published in the *Journal of the American Heart Association* examined the impact of flavored vape juice with nicotine (5 mg/mL) compared to a nicotine-free vehicle, inhaled over four weeks in mice subjected to acute myocardial infarction (MI). The results demonstrated significantly decreased myocardial function post-MI in mice exposed to nicotine aerosol, irrespective of sex—highlighting the deleterious role of nicotine on cardiac contractility [83]. In another study, rats exposed to either 12 weeks of purified air or e-cigarette vapor (15 mg/mL nicotine) during the post-MI healing phase showed altered vascular function, although no significant changes were observed in left ventricular dilation or cardiac function [84]. Even acute exposure to e-cigarettes has been associated with cardiovascular changes. Notably, increases in heart rate (HR) and blood pressure (BP) have been observed among users. While HR elevation was less pronounced with e-cigarettes than traditional cigarettes, systolic and diastolic BP differences were not statistically significant [85]. The formulation of nicotine in e-liquids may further influence cardiovascular outcomes. A recent murine study found that the inhalation of aerosols from nicotine-salt e-liquids (as opposed to racemic or free-base nicotine) increased the risk of cardiac arrhythmias by promoting sympathetic dominance [86]. An increasing body of epidemiological research indicates that e-cigarettes are an independent risk factor for cardiovascular disease (CVD) and mortality [82]. Meta-analyses have linked e-cigarette use to increased risks of arrhythmias and myocardial infarction (MI), primarily due to harmful aerosol constituents such as nicotine and flavorings [87]. Regular e-cigarette use, particularly among dual users (those who use both e-cigarettes and traditional cigarettes), was associated with a substantially elevated risk of MI. One meta-analysis found that dual use increased the odds of CVD by 2.56 times (95% CI: 2.11–3.11), and current e-cigarette use with former traditional smoking increased the odds by 2.02 times (95% CI: 1.58–2.58), compared to never-users. Interestingly, exclusive e-cigarette use did not show a statistically significant association with CVD (OR = 1.24, 95% CI: 0.93–1.67) [88]. A cross-sectional U.S. study further found that former and occasional e-cigarette users had 23% and 52% greater odds, respectively, of ever having experienced an MI compared to never-users (OR = 1.23, 95% CI: 1.08–1.40, *p* = 0.001; OR = 1.52, 95% CI: 1.10–2.09, *p* = 0.010) [89]. Given these findings, major health organizations have issued cautionary guidelines regarding e-cigarette use. The European Society of Cardiology and the European Association of Preventive Cardiology recommend that e-cigarettes only be used as a tool for smoking cessation within a structured clinical program [90,91]. Moreover, the World Health Organization, in its first clinical guideline on tobacco cessation, does not endorse e-cigarettes as a therapeutic option [92].

## 7. Perceptions and Behaviors Related to Vaping in Adolescents and Young Adults

Peer influence and sibling modeling are among the primary pathways through which adolescents and young adults are introduced to e-cigarette use. Curiosity, the desire for social acceptance, and a predisposition toward risk-taking—traits commonly associated with adolescence—serve as key motivators for initiating vaping. The pursuit of social belonging and the desire to fit in with peers further reinforce the uptake of e-cigarette use. Flavored e-liquids, with their appealing scents and tastes, play a substantial role in attracting adolescents and sustaining their engagement with vaping. These sensory features contribute to the widespread perception that e-cigarettes are less harmful than traditional tobacco products, thus normalizing the behavior within youth culture. In addition to recreational use, many adolescents report using e-cigarettes as a coping mechanism—to manage social anxiety, reduce stress, or self-soothe in challenging emotional situations. However, this perceived relief is often short-lived and may contribute to the development of nicotine dependence. Vaping addiction is a growing concern, particularly due to the high concentrations of nicotine in many e-cigarette products. As dependence increases, users often require progressively higher doses of nicotine to achieve the desired effect. In situations where e-cigarettes are unavailable, cravings may provoke intense emotional responses, which adolescents may struggle to regulate effectively. These experiences of withdrawal and emotional dysregulation often lead to failed cessation attempts and further entrench the cycle of dependence [93].

## 8. Youth Cessation Policies Regarding E-Cigarettes: Prevention and Intervention Strategies

**Figure 1:**
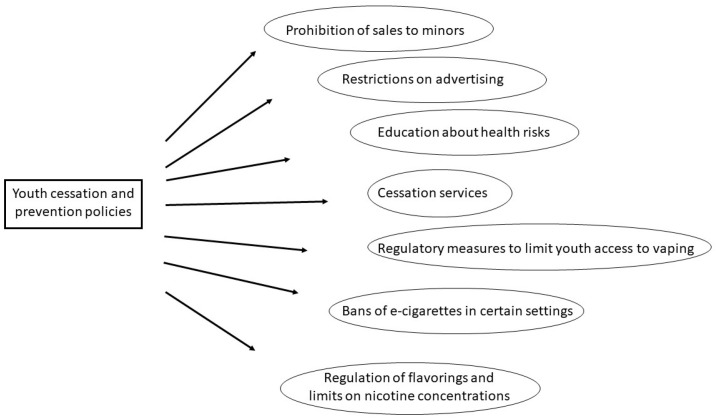
Youth cessation and prevention policies.

Cessation policies targeting youth, particularly in relation to e-cigarette use, focus on prevention and counteracting consumption. These efforts aim to restrict access, raise awareness of health risks, and support individuals who wish to quit. Key elements include the following: prohibition of sales to minors; restrictions on advertising; educating youth about the health risks associated with vaping and smoking; cessation services and resources for adolescents who have already started using e-cigarettes or conventional tobacco products; regulatory measures to limit youth access to vaping products and ensure safer standards in order to protect public health; bans of e-cigarettes in certain settings, such as schools, training centers, and public spaces; and the adoption of further restrictions, as some countries have carried out, such as the regulation of flavorings and limits on nicotine concentrations (Figure 1).

## 9. Effect of Vaping on Pregnancy and Fetus

An increasing number of pregnant women are switching from traditional cigarettes to electronic cigarettes (e-cigarettes), often under the perception that vaping is a safer alternative [94]. A recent meta-analysis reported that prenatal vaping is associated with a 53% increased risk of adverse maternal outcomes, particularly reduced breastfeeding rates and lower engagement with adequate prenatal care. Similarly, prenatal exposure to e-cigarettes was linked to a 53% increased risk of adverse neonatal outcomes, including low birth weight, preterm birth, and small-for-gestational-age status [95].

A case study investigating passive exposure to vaping during pregnancy demonstrated the transplacental transfer of several harmful substances, including glycerol, aluminum, chromium, nickel, copper, zinc, selenium, and lead, from the mother to the fetus via cord blood. Furthermore, in utero exposure to chemicals associated with Electronic Nicotine Delivery Systems (ENDSs) was detected in critical fetal organs such as the lungs, kidneys, brain, bladder, and heart [96]. Several studies using murine models have demonstrated that both nicotine-containing and nicotine-free e-cigarette aerosols adversely affect offspring respiratory health by increasing susceptibility to pulmonary diseases later in life [97]. Additionally, transcriptomic analysis of fetal lung tissue revealed substantial dysregulation in gene expression, indicating molecular alterations in response to prenatal vaping exposure [98]. Other preclinical studies have examined the effects of exposure to additives in nicotine-free e-cigarettes on postnatal neurological development in mice. These investigations identified increased long-term anxiety-like behavior and impaired locomotor activity in offspring, especially following late gestational and prolonged exposure [99]. These findings are corroborated by in vitro studies demonstrating the neurotoxic potential of e-cigarette vapor constituents on fetal brain development [100].

## 10. Conclusions

Electronic cigarettes and similar devices used to inhale heated and aerosolized substances have been promoted as a potentially less harmful and carcinogenic alternative to traditional combustible cigarettes, particularly for aiding heavy smokers in quitting. However, they have gained widespread popularity among young adults and adolescents—especially among individuals who have never smoked—largely due to the misconception that these products pose no health risks. Rather than serving as an effective cessation tool, e-cigarettes have, in many cases, acted as a gateway, encouraging nicotine use among never-smokers more often than they have supported smoking cessation among long-term users. A growing body of scientific evidence now implicates e-cigarette use in the development of various health conditions, most notably cancers, chronic obstructive pulmonary disease (COPD), asthma, e-cigarette or vaping-associated lung injury (EVALI), cardiovascular disease, and diffuse parenchymal lung diseases. Given these risks, leading medical organizations, including the European Society of Cardiology and the European Association of Preventive Cardiology, recommend that e-cigarettes be used solely as a tool for tobacco cessation, and only within the framework of a structured cessation program.

## Data Availability

Data are available on request.

## Abbreviations

The following abbreviations are used in this manuscript:

ENDS	Electronic Nicotine Delivery System
WHO	World Health Organization
THC	Tetrahydrocannabinol
EVALI	E-cigarette or Vaping Product Use-Associated Lung Injury
VEA	Vitamin E acetate
ROS	Reactive oxygen species
IL-8	Interleukin-8
COPD	Chronic obstructive pulmonary disease
CPAP	Continuous Positive Airway Pressure
MI	Myocardial infarction
